# Spotlight on Seniors with Narcolepsy: Comorbidities and Management

**DOI:** 10.3390/jcm14093217

**Published:** 2025-05-06

**Authors:** Rena Y. Jiang, Shae Duka, Martina Vendrame

**Affiliations:** 1 Lehigh Valley Fleming Neuroscience Institute, Lehigh Valley Health Network, Allentown, PA 18104, USA; jiang.rena.030@gmail.com; 2 Neurology Department, University of South Florida Morsani College of Medicine, Tampa, FL 33602, USA; 3 Network Office of Research and Innovation, Lehigh Valley Health Network, Allentown, PA 18104, USA; shae.duka@lvhn.org

**Keywords:** narcolepsy, cataplexy, excessive daytime sleepiness, multiple sleep latency test, sleep disorders, comorbidity, migraine, depression, hypertension, geriatric

## Abstract

**Background/Objectives**: Narcolepsy was first described in the late 19th century, and in the current decade, narcolepsy patients are reaching their senior years. Little is known about the evolution of clinical features, the management of narcolepsy medications, and the development of comorbid conditions. We aimed to present the clinical characteristics, comorbidities, and therapeutic choices of seniors with narcolepsy. **Methods**: We extracted 21 charts of patients older than 65 with a diagnosis of narcolepsy according to the International Classification of Sleep Disorders Third Edition. We reviewed and analyzed all clinical and available polysomnographic data. **Results**: A total of 21 patients (median age 69 years. 67.0–71.0 interquartile range IQR; 71% female) were included. Three (14.3%) had type I and 18 (85.7%) had type II narcolepsy. The average age at symptom onset was 23 years (IQR 19.5–27.5). Diagnosis was made at an average age of 41 years (IQR 33–45), between 1990 and 2002. Median time from onset to diagnosis was 13.7 years (IQR 9.5–19). The most prevalent cardiovascular/metabolic comorbidity was hypertension (57.1%). All patients were historically using narcolepsy medications. Fewer patients were currently on wake-promoting agents (85.7%), with over half on modafinil (55.6%). None currently reported the need to nap during the daytime. **Conclusions**: Narcolepsy is a lifelong, but not progressive disorder, that has yet to be well-characterized in the senior population. A few seniors appear to outgrow the disorder and to no longer need wake-promoting agents. It is important to consider cardiometabolic comorbidities in the management of narcolepsy in this population. Geriatricians should be educated on narcolepsy with specific programs for these seniors.

## 1. Introduction

Narcolepsy was first described in Germany in the late 19th century: Westphal and Fisher reported two cases of daytime sleepiness associated with episodes of muscle weakness brought on by excitement [1]. In the 1930s, Von Economo identified the posterior hypothalamus as a key region for maintaining wakefulness. He further hypothesized that a disease of that region underlies narcolepsy [1]. Treatment of narcolepsy first appeared in the form of intrathecal air injection, cerebrospinal fluid removal, and irradiation of the hypothalamic region [1]. Amphetamines were used to treat daytime sleepiness, and methylphenidate came onto the scene several decades later in the 1960s [1]. Soon after the discovery of tricyclic antidepressants, clinicians began using them to treat cataplexy [1]. This marks the establishment of the commonly used pharmacologic combination used for narcolepsy today.

Today, narcolepsy is known as a life-long disorder that presents primarily with excessive daytime sleepiness. This is thought to be due to insufficient activation of wake-promoting orexin neurons in the hypothalamus [2]. For most patients, symptom onset occurs in their early 20s [3]. Daytime sleepiness can be accompanied by other clinical features such as sleep paralysis, fragmented sleep, and hypnogogic hallucinations [2]. Narcolepsy is classified by the International Classification of Sleep Disorders Third Edition (ICSD-III) [4] into two types: type 1 (NT1) and type 2 (NT2). NT1 presents with cataplexy, whereas NT2 does not [2]. Cataplexy is triggered by strong emotions, often positive ones. It involves a loss of muscle tone that evolves over seconds. Significantly, breathing and consciousness are not affected in cataplexy [2]. Further differentiating the two types of narcolepsy, people with NT1 have low orexin levels in their cerebrospinal fluid, while people with NT2 have normal levels [2]. The cause of low orexin levels may have an autoimmune or heritable component [2]. The symptoms associated with NT1, such as daytime sleepiness, sleep paralysis, and sleep hallucinations, are generally more pronounced compared to those with NT2 [2]. The cause of NT2 is still unclear.

Narcolepsy is associated with comorbid conditions across several domains including neurologic, psychiatric, cardiovascular, metabolic, and sleep [5]. Prior studies have shown that headaches [6,7], depression [6,8,9,10], anxiety [6,7,9], hypertension [11,12], obesity [11,13], and sleep apnea [6,14] are common in people with narcolepsy. However, there is little known about how these comorbidities manifest specifically in senior populations with narcolepsy.

While narcolepsy typically first presents in young adulthood, it is a chronic but not disabling disorder. The establishment of the wake-promoting/antidepressant pharmacological treatment for narcolepsy in the 1960s and the development of the multiple sleep latency test (MSLT) in the next decade highlighted important components of narcolepsy management today [1]. In the current decade, we are seeing patients with narcolepsy reaching their senior years. Little is known about the evolution of clinical features, the management of narcolepsy medications, and the development of comorbid conditions in this demographic.

The scope of this study is to present the clinical characteristics, therapeutic choices, and comorbidities of seniors within the Lehigh Valley Health Network who carry a diagnosis of NT1 or NT2.

## 2. Materials and Methods

Institutional Review Board (IRB) approval was obtained before initiating the study (IRB study number 1297). We undertook an exploratory, descriptive, retrospective analysis of a small subgroup of seniors with narcolepsy seen in the Lehigh Valley Health Network between 2000 and 2022. The system database was queried for “narcolepsy”. All charts of patients with a diagnosis of “narcolepsy” and aged 65 and above were extracted and manually reviewed. All charts were reviewed by a board-certified sleep specialist to confirm accurate diagnosis and categorization into narcolepsy type 1 and narcolepsy type 2.

Data on demographics (age, gender, body mass index), narcolepsy specific clinical information (age at disease onset, age at diagnosis, presence and severity of daytime sleepiness, cataplexy, sleep paralysis, hypnagogic hallucinations, presence of disrupted sleep, need for restorative naps), Epworth Sleepiness Scale (ESS) scores, data on narcolepsy testing including polysomnography (PSG) and multiple sleep latency test (MSLT), narcolepsy specific medications (wake-promoting and anti-cataplexy agents), sleep comorbidities (insomnia, sleep related breathing disorders, parasomnias, sleep related movement disorders), psychiatric disorders, cardiovascular disorders and metabolic disorders were collected.

All study data were recorded and maintained in REDCap (Research Electronic Data Capture)—a secure, web-based software platform designed to support data capture for research studies [15,16]. Patients who did not meet the current ICSD-III [4] diagnostic criteria for narcolepsy type 1 or narcolepsy type 2 were excluded.

ICSD-III diagnostic criteria for narcolepsy type 1 include both of the following: (1) excessive daytime sleepiness daily for three months or more; (2) cataplexy and mean sleep latency less than or equal to 8 min and greater than or equal to two sleep onset rapid eye movement periods on MSLT, OR low cerebrospinal fluid (CSF) orexin levels [4]. Of note, none of the patients included in this study had CSF orexin levels examined.

ICSD-III diagnostic criteria for narcolepsy type 2 include all of the following: (1) excessive daytime sleepiness and MSLT criteria as in type 1 criteria, but without cataplexy; (2) CSF orexin levels are unknown or above threshold for narcolepsy type 1; (3) sleepiness and MSLT findings are not better explained by other causes such as sleep deficit, other sleep disorder, medication use or medication withdrawal [4].

Descriptive statistics were conducted to summarize the demographics, narcolepsy related clinical variables, comorbidities, and polysomnographic features of the entire study sample. Continuous variables were reported as the mean and standard deviation (SD) or the median and interquartile range (IQR), depending on normality. Categorical variables were presented as frequency and percent.

## 3. Results

### 3.1. Demographic and Clinical Characteristics

Table 1 shows key demographic and clinical features of each patient in this study. A total of 21 (21/304, 6.9%) patients were included in the study. Three (14.3%) had NT1 and 18 (85.7%) had NT2 (Table 1; Table 2). The average age at symptom onset was 23 years (IQR 19.5–27.5 years), and age at diagnosis was 41 years (IQR 33–45 years). Median time from onset to diagnosis was 13.7 years (IQR 9.5–19 years). The majority of the patients in the sample were female (71.4%), white/Caucasian (100%), and non-Hispanic or Latino (90.5%). The most common narcolepsy symptoms at presentation (outside of excessive daytime sleepiness) were a need for restorative sleep (71.4%), abruptly falling asleep (33.3%), disrupted sleep (33.3%), and hypnagogic hallucinations (28.6%).

The median ESS at diagnosis was 16.5 (IQR 15.0–20.0). At the most recent follow up visits, 12 (57%) out of the 21 patients reported symptoms of daytime sleepiness, some in conjunction with disrupted sleep and/or abruptly falling asleep during the daytime. None of the patients reported the need to take scheduled daytime naps. Recent ESS scores were available only for nine patients and ranged between 9 and 15 (Table 1).

### 3.2. Comorbidities

Cardiovascular and metabolic disorders were the most prevalent comorbidities in this cohort. Table 3 presents comorbidities (neurological, sleep, psychiatric, and cardio-metabolic disorders) and medication use within the entire sample. The most prevalent neurological comorbidity was headaches/migraines (23.8%), followed by stroke (19.1%) and traumatic brain injury (14.3%). Per chart review, while headaches/migraine were present in the peri-diagnostic period, stroke and traumatic brain injury occurred decades after the diagnosis of narcolepsy. The most common sleep comorbidity was breathing disorders such as obstructive sleep apnea (28.6%), which was also diagnosed later in life. Most patients did not have a psychiatric comorbidity but about one third of the patients had been treated for depression at some point. The most common cardiovascular/metabolic comorbidity was hypertension (57.1%). Other cardio-metabolic comorbidities included hyperlipidemia (38.1%), diabetes (33.3%) and obesity (19.1%); all other conditions were <15%. Almost all patients were on some pharmacological management for a comorbid condition (85.7%), with antihypertensives (61.1%) and diabetes medication (38.9%) being the most used.

### 3.3. Medical and Behavioral Management

All patients were historically using narcolepsy medications, with most patients using Modafinil (61.9%) (Table 3). Fewer patients were currently using wake-promoting agents (85.7%), with over half on Modafinil (55.6%) and Armodafinil (11.1%). One patient with NT2 was currently treated with Sodium Oxybate together with Modafinil. One patient with NT1 was currently on Soriamfetol and Venlafaxine for control of cataplexy. None of the patients currently reported the need to nap during the daytime and therefore there were no behavioral plans implemented.

### 3.4. PSG/MSLT Data

All 21 patients had an MSLT conducted and results were available for review. However, specific MSLT data were missing at large. Average sleep latency was 4.2 min, sleep time 17.2 min, sleep onset rapid eye movement periods (SOREMPs) 8 min, and 85.7% of patients had two naps with SOREMPs. Although a PSG was done for all patients, specific findings were not available for most patients. Periodic leg movements and bruxism were found for one patient. The median number of central apnea, obstructive apnea, mixed apnea, and hypopnea events were 0.

## 4. Discussion

We describe a cohort of seniors aged 65 and above with a diagnosis of narcolepsy type 1 or type 2 currently being followed in the Lehigh Valley Health Network. These patients presented with symptoms of narcolepsy (including the need for scheduled naps in the majority) in their twenties and had an average diagnostic delay of about 13 years. With MSLT emerging only in the 1980s, such a delay in this cohort is expected. Increased MSLT availability and awareness have reduced the current diagnostic delay across the globe to about 3–4 years [2,5,17,18]. Nevertheless, there have been reported cases of narcolepsy being diagnosed in the later decades of life [19]. In certain cases, it has been hypothesized that hospitalization for comorbid medical conditions may unmask previously unrecognized cataplexy later in life, thereby drawing clinical attention to long-standing symptoms of narcolepsy.

All the patients in our cohort were treated with wake-promoting agents at some point in their life, although it seems that only 86% of these patients remained on medications during their senior years. More than half of the cohort was currently on Modafinil or Armodafinil. The decision to stop wake-promoting agents may be multifactorial. As patients approach retirement age, they may not have the same functional requirements throughout the day. The severity of their narcolepsy symptoms may have also substantially decreased. Finally, given concurrent comorbidities, the cost–benefit of taking long-term stimulants may no longer align with the patients’ health priorities.

Of the three patients with NT1, only one was still on medical management with Venlafaxine, with currently no episodes of cataplexy. The natural prognosis of cataplexy in humans is unknown. Previous studies have shown mixed data on cataplexy frequency across age groups—some report fewer cataplexy events in seniors compared to younger populations [20,21], whereas others report no difference in cataplexy with age [22,23]. Concurrent medications, such as alpha blockers [24] and D2 agonists [25], can also exacerbate cataplexy across the lifespan [19]. In rodent models of narcolepsy, evidence shows that the narcolepsy/cataplexy phenotype caused by orexin deficiency in mice may be substantially preserved with aging [26]. In our cohort, the observation that two patients did not require treatment for cataplexy and remained asymptomatic may also suggest that their initial presentation did not represent true cataplexy. This highlights the importance of recognizing that cataplexy is primarily a clinical diagnosis, which inherently presents diagnostic challenges [2].

A review of most recent reported narcolepsy symptoms showed that only about half of the patients complained of excessive daytime sleepiness. Recent ESS scores were available only for nine patients and showed lower values in comparison to respective prior scores at diagnosis. Interestingly, none of these patients reported the need to nap during the daytime. It is well established that taking naps is a frequent practice of seniors [27], and although overall sleep quantity decreases with aging, the levels of nap-taking may even increase after age 65 [28]. Although we may argue that the sleep need may decrease with aging, it has to be taken into account that the majority of patients in the current cohort were currently treated with a wake-promoting agent on a daily basis.

In our cohort, 33% of patients complained of disrupted sleep at diagnosis and some reported difficulty with sleep maintenance later in life as well. In the general population, age-related disruption of nocturnal sleep is commonly attributed to progressive alterations in circadian regulation [27,29]. Some studies have demonstrated that individuals with narcolepsy may exhibit similar age-related sleep disturbances [20,29], whereas others have reported no significant differences in sleep architecture across age groups [22]. Studies in mouse models of narcolepsy have shown that aging mice develop progressive orexin neuron loss, and subsequent hyperexcitability of the remaining neurons occurs as a mode of circuit-based compensation [30]. Li et al. showed that narcoleptic mice also develop increasingly fragmented sleep, and this may be related to neural circuit malfunction or reorganization in the setting of rapid orexin neuron loss [30]. The exact mechanism remains to be elucidated.

While sleep fragmentation and decreased efficiency usually lead to increased daytime sleepiness—and thus, napping—in healthy adults [27,29], this relationship is not as clear in narcolepsy patients. Excessive daytime sleepiness, as measured by patient-reported outcomes (e.g., ESS) and objective sleep latency on the MSLT, has been reported to remain stable across the lifespan in some studies [20,21,29]. However, other investigations have shown variability with age [22,31]. These findings suggest that the underlying mechanisms of daytime sleepiness in older adults with narcolepsy may differ from those seen in the general aging population.

Analysis of comorbidities showed that hypertension was the most common comorbid medical disorder, with about 61% of patients using anti-hypertensive medications. This prevalence is similar to the one expected in the general senior population—57% in women, and 54% in men [32]. Long-term use of Modafinil can substantially perturbate autonomic cardiovascular regulation, causing a significant increase in blood pressure and heart rate [33]. Diabetes was the second most common cardiometabolic comorbidity among our patients. Similar to our finding, a previous report has shown an increased prevalence of hypertension and diabetes in senior narcolepsy patients compared to age-matched controls without narcolepsy [23].

Breathing disorders were the most common sleep disorder comorbidity, affecting about one third of the cohort. In addition, 70% of our senior narcolepsy patients were found to be in the overweight or obese BMI class. Of note, BMI is a risk factor for breathing disorders such as obstructive sleep apnea [34]. It may be prudent to consider the presence of a comorbid sleep breathing disorder in senior patients reporting daytime sleepiness, even when they have a pre-existing diagnosis of narcolepsy.

The strengths of our study include capturing a wide range of demographic and clinical variables on an infrequently studied group of narcolepsy patients. Our study only included patients with confirmed NT1 or NT2 diagnosis by MSLT and based on ICSD-III criteria, which eliminates potential errors that may arise from collecting narcolepsy cases from billing codes.

This study is significantly limited by its retrospective design, resulting in a lack of critical clinical detail, such as disease severity, duration of symptoms, and patient adherence to treatment. The study is also vulnerable to selection bias, as it included a population who may follow-up in the clinic exclusively for medication management. This means that patients with a diagnosis of narcolepsy, but who are off medications, may have been missed. To address these gaps, prospective studies and narcolepsy-specific registries are essential, as they will enable systematic and standardized data collection, improve capture of clinical variables, and support more robust and generalizable findings for the senior population.

As patients with narcolepsy are reaching senior age, it is essential to establish dedicated geriatric clinics for these patients. We have observed that older adults with narcolepsy often have multiple comorbidities, including cardiovascular and psychiatric conditions, which can complicate treatment. Geriatricians must be educated on the medical management of narcolepsy, including appropriate pharmacologic options, potential drug interactions, and age-related changes in sleep architecture and medication metabolism. Integrating narcolepsy care into geriatric practice will not only improve symptom control and quality of life, but also ensure that comorbid conditions are managed holistically and safely in this vulnerable population.

## Figures and Tables

**Table 1:** Demographics and Clinical Information of Each Patient.

**NO.**	**Age (Years)**	**Age of Onset (Years)**	**Age of** **Diagnosis (Years)**	**Narcolepsy Type**	**Narcolepsy** **Symptoms**			**ESS (0–24)**	**Gender**	**BMI**	**Neuro Disorders**	**Sleep Disorders**
1	71	22	34	NT2	Excessive Daytime Sleepiness, Abruptly Falling Asleep, Sleep Paralysis, Hypnagogic Hallucinations, Need for Restorative Sleep			21	F	21.51	Stroke, HA/Migraine	None
2	69	21	31	NT2	Excessive Daytime Sleepiness, Abruptly Falling Asleep, Difficulty Falling Asleep at Night, Presence of Disrupted Sleep, Need for Restorative Sleep			N/A	F	22.84	HA/Migraine	PLMD
3	84	19	24	NT2	Excessive Daytime Sleepiness, Abruptly Falling Asleep			15	F	N/A	None	None
4	78	29	45	NT1	Excessive Daytime Sleepiness, Abruptly Falling Asleep, Sleep Paralysis, Presence of Disrupted Sleep			21	F	27.77	None	None
5	70	23	42	NT2	Excessive Daytime Sleepiness, Need for Restorative Sleep			20	F	22.09	TBI, Stroke	None
6	66	25	41	NT2	Excessive Daytime Sleepiness, Hypnagogic Hallucinations, Need for Restorative Sleep			15	M	38.06	None	None
7	71	26	47	NT2	Excessive Daytime Sleepiness, Abruptly Falling Asleep, Sleep Paralysis, Hypnagogic Hallucinations, Need for Restorative Sleep			19	F	25.71	None	None
8	70	17	37	NT1	Excessive Daytime Sleepiness, Sleep Paralysis, Hypnagogic Hallucinations, Need for Restorative Sleep			17	F	32.94	None	Breathing disorders
9	66	23	33	NT2	Excessive Daytime Sleepiness, Need for Restorative Sleep			15	F	35.97	Stroke, HA/Migraine	Breathing disorders
10	68	29	35	NT2	Excessive Daytime Sleepiness			15	M	36.69	TBI, Stroke, HA/Migraine	Breathing disorders
11	66	N/A	57	NT2	Excessive Daytime Sleepiness			13	M	28.73	TBI, HA/Migraine	Insomnia, Breathing disorders
12	67	19	23	NT1	Excessive Daytime Sleepiness, Cataplexy, Sleep Paralysis, Hypnagogic Hallucinations, Hypnopompic Hallucinations, Presence of Disrupted Sleep, Need for Restorative Sleep			16	M	26.99	None	None
13	67	32	41	NT2	Excessive Daytime Sleepiness, Presence of Disrupted Sleep, Need for Restorative Sleep			16	F	22.83	None	Breathing disorders, RLS
14	68	19	30	NT2	Excessive Daytime Sleepiness, Abruptly Falling Asleep, Need for Restorative Sleep			16	F	19.92	None	None
15	69	20	47	NT2	Excessive Daytime Sleepiness, Abruptly Falling Asleep, Need for Restorative Sleep			14	F	27.76	None	None
16	67	24	43	NT2	Excessive Daytime Sleepiness, Presence of Disrupted Sleep, Need for Restorative Sleep			17	F	25.24	None	Insomnia
17	74	26	30	NT2	Excessive Daytime Sleepiness, Need for Restorative Sleep			16	F	24.54	None	None
18	75	33	49	NT2	Excessive Daytime Sleepiness, Hypnagogic Hallucinations, Presence of Disrupted Sleep			21	F	31.1	None	Insomnia
19	69	16	42	NT2	Excessive Daytime Sleepiness			18	F	29.27	None	RLS
20	78	35	45	NT2	Excessive Daytime Sleepiness, Presence of Disrupted Sleep, Need for Restorative Sleep			22	M	29.42	None	Insomnia
21	69	22	38	NT2	Excessive Daytime Sleepiness, Need for Restorative Sleep			20	M	31.09	None	Breathing disorders, RLS
**NO.**	**Psych Disorders**	**Cardio-** **Metabolic Disorders**		**Non-Narcolepsy Meds**	**History of Narcolepsy Meds**	**Current Narcolepsy Meds**	**History of Cataplexy Meds**	**Current Cataplexy Meds**		**Current Narcolepsy Symptoms**		**ESS (0–24) at Latest Visit**
1	MDD	HTN, CHF/CM		Antidepressants, Antianxiety, Antiplatelet, Anticoagulants, HA/Migraine Med, Other	Modafinil	Modafinil, Methylphenidate	None	None		Excessive Daytime Sleepiness, Abruptly Falling Asleep		12
2	MDD	None		Antidepressants, HA/Migraine Med	Modafinil	Modafinil	None	None		Excessive Daytime Sleepiness, Difficulty Falling Asleep at Night		N/A
3	None	HTN		Anti-HTN	Methylphenidate	Methylphenidate	None	None		None reported		N/A
4	None	HTN		Anti-HTN	Modafinil	None	None	None		None reported		N/A
5	None	HTN, DM, CHF/CM, HLD		Anti-HTN, DM Med, Anticoagulants, Other	Armodafinil	Armodafinil	None	None		Excessive Daytime Sleepiness		11
6	None	HTN, DM		Anti-HTN, DM Med	Modafinil	Methylphenidate	None	None		Excessive Daytime Sleepiness		N/A
7	None	DM		DM Med	Modafinil	Modafinil	None	None		None reported		N/A
8	None	HTN, DM, Obesity, HLD		Anti-HTN, DM Med, HA/Migraine Med	Modafinil, Armodafinil	Modafinil	None	None		None reported		14
9	Anxiety, MDD	HTN, DM, Obesity, CHF/CM, HLD, Arrhythmia		DM Med, HLD Med	Modafinil	None	None	None		Excessive Daytime Sleepiness		13
10	MDD	HTN, Obesity		Antidepressants, Anti-HTN, Antiplatelet, HLD Med	Modafinil, Armodafinil, Dextroamphetamine, Sodium Oxybate, Pitolisant, Solriamfetol	Modafinil, Dextroamphetamine	None	None		Excessive Daytime Sleepiness		11
11	None	HTN, HLD, Arrhythmia		Anti-HTN, Anticoagulants	Armodafinil, Methylphenidate	None	None	None		Excessive Daytime Sleepiness		N/A
12	None	None		None	Dextroamphetamine	Solriamfetol	Venlafaxine	Venlafaxine		Excessive Daytime Sleepiness, Cataplexy		N/A
13	None	None		None	Modafinil, Methylphenidate	Methylphenidate	None	None		None reported		N/A
14	None	None		None	Sodium Oxybate	Modafinil	None	None		Excessive Daytime Sleepiness, Abruptly Falling Asleep		15
15	None	HLD		HLD Med	Methylphenidate, Dextroamphetamine	Modafinil	None	None		None reported		10
16	MDD	None		Antidepressants	Modafinil, Sodium Oxybate	Modafinil, Sodium Oxybate	None	None		Excessive Daytime Sleepiness, Presence of Disrupted Sleep		N/A
17	None	Htn, Hypothyroidism		Anti-HTN, Other	Methylphenidate	Methylphenidate	None	None		None reported		N/A
18	Anxiety, MDD, SCZ/Psychotic Disorders	HTN, HLD		Antidepressants, Antianxiety, Anti-HTN, Antiplatelet, HLD Med	Modafinil	Modafinil	None	None		Excessive Daytime Sleepiness, Presence of Disrupted Sleep		9
19	None	HTN, DM, HLD		Anti-HTN, DM Med	Modafinil, Dextroamphetamine	Armodafinil	None	None		None reported		N/A
20	Anxiety, MDD	None		Antianxiety	Methylphenidate	Methylphenidate	None	None		Excessive Daytime Sleepiness, Presence of Disrupted Sleep		N/A
21	None	DM, Obesity, HLD		Anti-HTN, DM Med, Anticoagulants, HLD Med	Modafinil	Modafinil	None	None		None reported		11

M = male. F = female. N/A = not available. TBI = traumatic brain injury. HA = headache. PLMD = periodic leg movement disorder. MDD = major depressive disorder. SCZ = schizophrenia. HTN = hypertension. DM = diabetes mellitus. CHF = congestive heart failure. CM = cardiomyopathy. HLD = hyperlipidemia. Med = medication. ESS = Epworth Sleepiness Scale.

**Table 2:** Summary of Demographics and Clinical Information.

Variable	All Seniors (*n* = 21)
Age, years median (IQR)	69.0 (67.0–71.0)
Age at onset, years median (IQR) (*n* = 20)	23.0 (19.5–27.5)
Age at onset (Cat.) N (%) (*n* = 20)	
0–18	2 (10.0)
19–29	15 (75.0)
30–39	3 (15.0)
40–49	0
50–59	0
Age at diagnosis, years median (IQR)	41.0 (33.0–45.0)
Age at diagnosis (Cat.) *n* (%)	
0–18	0
19–29	2 (9.5)
30–39	8 (38.1)
40–49	10 (47.6)
50–59	1 (4.8)
Time from onset to diagnosis, years median (IQR) (*n* = 20)	13.7 (9.5–19.0)
Gender *n* (%)	
Male	6 (28.6)
Female	15 (71.4)
Race *n* (%)	
White/Caucasian	21 (100)
Ethnicity *n* (%)	
Hispanic or Latino	2 (9.5)
Non-Hispanic or Latino	19 (90.5)
Weight, kg median (IQR)	78.0 (63.5–93.4)
Height, cm median (IQR) (*n* = 20)	170.0 (160.7–177.7)
BMI, kg/m^2^ median (IQR) (*n* = 20)	27.8 (23.7–31.1)
BMI Class *n* (%) (*n* = 20)	
Underweight	0
Normal/Healthy	6 (30.0)
Overweight	8 (40.0)
Obese	6 (30.0)
Narcolepsy Symptoms at presentation	
Excessive Daytime Sleepiness	21 (100)
Abruptly Falling Asleep	7 (33.3)
Cataplexy	1 (4.8)
Sleep Paralysis	5 (23.8)
Hypnagogic Hallucinations	6 (28.6)
Hypnopompic Hallucinations	1 (4.8)
Difficulty Falling Asleep at Night	1 (4.8)
Presence of Disrupted Sleep	7 (33.3)
Need for Restorative Sleep	15 (71.4)
Other	0
Epworth Sleepiness Score (0–24) median (IQR) (*n* = 20) at diagnosis	16.5 (15.0–20.0)

IQR = interquartile range. kg = kilograms. cm = centimeters. m = meters. BMI = body mass index. Categorical variables are presented as the frequency (*n*) and percentage (%). Percentages are based upon column totals, unless the *n* for a given variable changes (e.g., age at onset). Continuous variables are presented as the median and interquartile range due to the small sample size.

**Table 3:** Comorbidities and Medications.

Variable	Senior Patients (*n* = 21)
Neurological Disorders *n* (%)	
Traumatic Brain Injury	3 (14.3)
Stroke	4 (19.1)
Parkinson’s Disease	0
Headache/Migraines	5 (23.8)
Seizures/Epilepsy	0
Brain Tumor	0
Multiple Sclerosis	0
Other	0
None	15 (71.4)
Sleep Disorders *n* (%)	
Insomnia	4 (19.1)
Breathing Disorders	6 (28.6)
Non-REM Parasomnias	0
Periodic Limb Movement Disorder	1 (4.8)
RLS	3 (14.3)
REM Parasomnia	0
Circadian Rhythm Sleep–Wake Disorders	0
Other	0
None	10 (47.6)
Psychiatric Disorders *n* (%)	
Anxiety	3 (14.3)
Major Depressive Disorder	7 (33.3)
Schizophrenia/Psychotic Disorders	1 (4.8)
None	14 (66.7)
Cardiovascular/Metabolic Disorders *n* (%)	
Hypertension	12 (57.1)
Diabetes	7 (33.3)
Obesity	4 (19.1)
CHF/Cardiomyopathy	3 (14.3)
Hyperlipidemia	8 (38.1)
Arrhythmia	2 (9.5)
Other	1 (4.8)
None	6 (28.6)
Non-Narcolepsy Medication Use *n* (%)	18 (85.7)
Antidepressants	5 (27.8)
Antianxiety	3 (16.7)
Antipsychotics	0
Antihypertensives	11 (61.1)
DM Medication	7 (38.9)
Antiplatelet	3 (16.7)
Anticoagulants	4 (22.2)
Hyperlipidemia Medication	5 (27.8)
Headache/Migraine Medication	3 (16.7)
Anticonvulsants	0
Multiple Sclerosis Medication	0
Dopaminergic (Parkinsons)	0
Other	3 (16.7)
History of Narcolepsy Medication Use *n* (%)	21 (100)
Modafinil	13 (61.9)
Armodafinil	4 (19.1)
Methylphenidate	6 (28.6)
Dextroamphetamine	4 (19.1)
Sodium Oxybate	3 (14.3)
Pitolisant	1 (4.8)
Solriamfetol	1 (4.8)
Other	0
Current Narcolepsy Medication Use *n* (%)	18 (85.7)
Modafinil	10 (55.6)
Armodafinil	2 (11.1)
Methylphenidate	6 (33.3)
Dextroamphetamine	1 (5.6)
Sodium Oxybate	1 (5.6)
Pitolisant	0
Solriamfetol	1 (5.6)
Other	0
History of Cataplexy Medication Use *n* (%)	1 (4.8)
Venlafaxine	1 (100)
Current Cataplexy Medications *n* (%)	1 (4.8)
Venlafaxine	1 (100)

REM = rapid eye movement. RLS = restless leg syndrome. CHF = congestive heart failure. DM = diabetes mellitus. Categorical variables are presented as the frequency (*n*) and percentage (%). Percentages are based upon column totals, unless the *n* for a given variable changes (e.g., narcolepsy medication use).

## Data Availability

The original contributions presented in this study are included in the article. Further inquiries can be directed to the corresponding author.

## Abbreviations

The following abbreviations are used in this manuscript:

BMI	body mass index
CHF	congestive heart failure
CSF	cerebrospinal fluid
DM	diabetes mellitus
ESS	Epworth Sleepiness Scale
ICSD-III	International Classification of Sleep Disorders Third Edition
IQR	interquartile range
IRB	Institutional Review Board
LVHN	Lehigh Valley Health Network
MSLT	multiple sleep latency test
NT1	narcolepsy type 1
NT2	narcolepsy type 2
PSG	polysomnography
REDCap	Research Electronic Data Capture
REM	rapid eye movement
RLS	restless leg syndrome
SD	standard deviation
SOREMPs	sleep onset rapid eye movement periods
